# Resistive Switching of GaAs Oxide Nanostructures

**DOI:** 10.3390/ma13163451

**Published:** 2020-08-05

**Authors:** Vadim Avilov, Nikita Polupanov, Roman Tominov, Maxim Solodovnik, Boris Konoplev, Vladimir Smirnov, Oleg Ageev

**Affiliations:** 1 Institute of Nanotechnologies, Electronics and Electronic Equipment Engineering, Southern Federal University, 347922 Taganrog, Russia; avilovvi@sfedu.ru (V.A.); npolupanov@sfedu.ru (N.P.); tominov@sfedu.ru (R.T.); solodovnikms@sfedu.ru (M.S.); kbg@sfedu.ru (B.K.); ageev@sfedu.ru (O.A.); 2 Research and Educational Centre “Nanotechnologies”, Southern Federal University, 347922 Taganrog, Russia

**Keywords:** atomic force microscopy, local anodic oxidation, gallium arsenide, oxide nanoscale structure, profiled nanoscale structure, effect of resistive switching

## Abstract

The paper presents the results of experimental studies of the influence of the local anodic oxidation control parameters on the geometric parameters of oxide nanoscale structures (ONS) and profiled nanoscale structures (PNS) on the surface of epitaxial structures of silicon doped gallium arsenide with an impurity concentration of 5 × 10^17^ cm^−3^. X-ray photoelectron spectroscopy measurements showed that GaAs oxide consists of oxide phases Ga_2_O_3_ and As_2_O_3_, and the thickness of the Ga_2_O_3_ layer is 2–3 times greater than the thickness of As_2_O_3_ area—i.e., the oxidized GaAs region consists mainly of Ga_2_O_3_. The experimental studies of the influence of ONS thickness on the resistive switching effect were obtained. An increase in the ONS thickness from 0.8 ± 0.3 to 7.6 ± 0.6 nm leads to an increase in the switching voltage *Uset* from 2.8 ± 0.3 to 6.8 ± 0.9 V. The results can be used in the development of technological processes for the manufacturing of nano-electronic elements, such as ReRAM, as well as a high-efficiency quantum dot laser.

## 1. Introduction

Nanotechnology in electronics has become one of the most promising areas of research that can bring significant progress in the development of new generation devices [1,2,3]. In particular, the physical implementation of non-volatile resistive access memory (ReRAM) has made great progress in the development of new generation computer memory and neuromorphic systems [4,5]. The ReRAM element (memristor) is an oxide layer between two conductive contacts and is based on the effect of resistive switching, a change in the electrical resistance of the oxide between the high resistance state (HRS) and low resistance state (LRS) under the influence of an external electric field [6]. The change in resistance is due to the formation and destruction of the nanoscale conduction channel, consisting of oxygen vacancies [7]. It is important to note that the manufacture of memristor structures is associated with the development and study of methods for modifying the substrate surface with high reproducibility and spatial resolution, including at the prototyping stage. In this case, methods that do not require labor-intensive technological operations associated with the preparation of special patterns and the application of resistive masks are of particular interest. There is a wide range of such methods (nanoimprinting [8,9], electron beam lithography [10,11], focused ion beams [12,13]), each of which has its own advantages and disadvantages associated with productivity, processing area, cost, etc., and cannot be considered ideal and generally accepted [14].

Lithography using scanning probe microscopy (SPM) is a promising method for the formation and prototyping of nanoscale structures and has several advantages, such as in situ control, the absence of radiation defects, and the absence of the need of produce expensive templates. One of the types of SPM lithography is local anodic oxidation (LAO), the essence of which is the phenomenon of electrolysis that occurs between an AFM probe and a conducting sample under the influence of an external electric field [15,16,17,18]. In this case, a water meniscus is used as the working medium, which is naturally formed between the AFM probe and the sample under atmospheric conditions [19,20,21,22].

The LAO method allows for the formation of oxide nanoscale structures (ONS) both on the surface of metals and on the surface of epitaxial semiconductor structures. Thus, ONS can be used for the manufacturing of resistive memory elements, quantum dot contacts, single-electron transistors, quantum wires, graphene nanoribbons [23,24,25,26,27].

One of the most promising materials for nanoelectronics is the GaAs epitaxial layers, which are widely used in the creation of integrated circuits that operate at frequencies above 10 GHz, microwave devices with low noise, high-efficiency quantum dot lasers [28,29], elements of multispectral quantum optoelectronics, [30,31], high-density storage devices [32], and solid-state biological hybrid technologies [33]. Moreover, in [34] a memristor effect is presented in thin films of gallium oxide.

After the chemical etching of ONSs, formed on the surface of GaAs epitaxial structure by the LAO method, it is possible to create profiled nanoscale structures (PNSs) as a centers for localizing quantum dots growth [35,36,37,38], nanowires [39,40], and also antilattices used in the development of the element base of quantum computers [41]. For the manufacture of such structures, it is necessary to form ONS and then PNS on the GaAs surface with specified geometric parameters. It was shown in [15,16,17,18,19,20,21,22] that the following control parameters of LAO mainly influence the height and diameter of GaAs ONS: the amplitude and duration of voltage during LAO, relative humidity inside the process chamber, material and pressure force of the AFM probe to the sample surface. It is also worth noting that, for ReRAM elements manufacturing, it is important to accurately control the thickness of the oxide structure, since the stability of the memristor elements depends on the scatter of their values. Therefore, the study of the depth of the PNS is an urgent task. However, at present, the influence of LAO regimes on the geometric parameters of PNS on the surface of GaAs epitaxial layers remains poorly studied.

Thus, the aim of the work is to study the influence of the amplitude and duration of voltage pulses during LAO relative humidity, and the intensity of the interaction of the AFM probe with the sample surface on the geometric parameters of the ONS and PNS on the surface of gallium arsenide. Additionally, the purpose of this work is to study the effect of the resistive switching of fabricated ONS.

## 2. Materials and Methods

To study the influence of the LAO regimes on the nanoscale profiling of GaAs surface, experimental samples were grown in a STE35 MBE system (SemiTEq, Saint-Petersburg, Russia) with solid phase sources. The structures were grown on semi-insulating GaAs (100) epi-ready wafers. After the thermal removal of the native oxide, a 500 nm thick GaAs buffer layer was grown. Then, under standard conditions (growth temperature is 580 ℃, growth rate is 1 μm/h), an n-type GaAs epitaxial layer with a thickness of 500 nm was formed and doped with silicon at a concentration of 5 × 10^17^ cm^−3^. This doping level was chosen in order to provide a low resistance of the GaAs epitaxial layer (0.001 Ohm∙cm, according to measurements by the Hall method), which allows for the formation of oxide structures at lower voltages and, as a result, make it more controllable.

Gallium arsenide oxide nanoscale structures were formed using the Ntegra probe nanolaboratory (NT-MDT, Moscow, Russia) by local anode oxidation in the semi-contact mode of atomic force microscope (AFM). For the LAO process, cantilevers NSG10 with Pt coating were used. LAO was carried out with the following technological parameters: the relative humidity of the atmosphere inside the technological SPM chamber was 70 ± 1% and 90 ± 1%; the feedback loop current (SetPoint parameter) varied from 0.1 to 5 nA; the amplitude and duration of voltage pulses in the probe-substrate system varied from 6 to 15 V, and from 100 to 1000 ms, respectively. Humidity was monitored using an ETHG913R digital moisture meter (Oregon Scientific, Portland, OR, USA).

**Figure 1:**
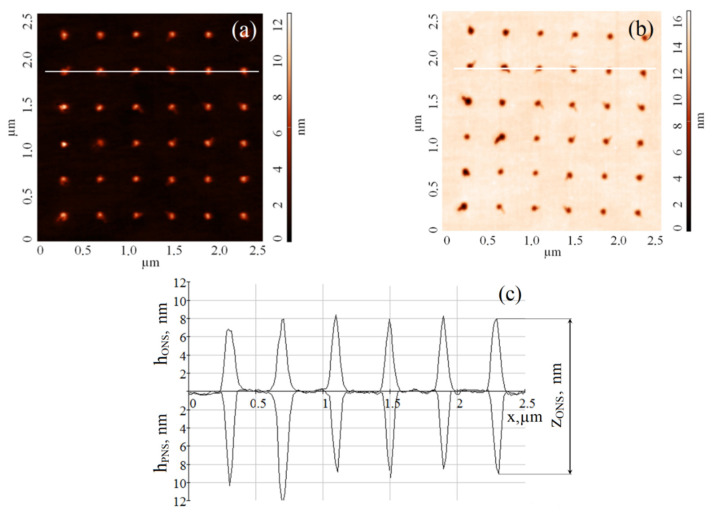
AFM image of the GaAs surface: (**a**)—after the formation of ONS by the LAO method; (**b**)—after the formation of the PNS; (**c**)—AFM cross-section along the lines on a and b.

As a result, 18 arrays of 72 islet GaAs ONSs each were formed on the gallium arsenide epitaxial structure surface (Figure 1a). To obtain PNS on the surface of gallium arsenide epitaxial structure, the obtained ONS were exposed to chemical etching in a solution of NH_4_OH (1:3) for 10 s. The obtained gallium arsenide PNS were studied by using Ntegra in the semi-contact mode (Figure 1b,c).

For statistical processing of the obtained AFM images, the Image Analysis 3.5 software package was used. Based on the statistical data obtained, the dependences of the geometric parameters of the ONS (height and diameter) and PNS (depth and diameter) on the amplitude, pulse duration of the applied voltage, and the SetPoint parameters were plotted for the relative humidity of 70 ± 1% and 90 ± 1%.

The study of the oxide structures composition was carried out by X-ray photoelectron spectroscopy (XPS) method using an Escalab 250 system (Thermo Scientific, Waltham, MA, USA). The XPS spectra were excited by monochromatized radiation from the AlKα line (hn = 1486.6 eV). Spectra were recorded on a GaAs surface section modified by the LAO method. The size of the modified surface was 300 × 300 μm^2^, and the diameter of the analyzed area (X-ray spot) on the surface of the sample was 250 μm. The elemental composition of the samples was determined from survey spectra taken at an analyzer transmittance of 150 eV. Individual lines (C1s, O1s, Ga3d, As3d) were recorded at an analyzer transmittance of 20 eV. The spectrometer was preliminarily calibrated by the binding energies of the Ag3d5/2 (368.2 eV) and Au4d (84.0 eV) lines obtained from the metal surfaces after they were cleaned with Ar ions. The minimum resolved energy range, determined by the full width at half the height of the Ag3d5/2line, was no worse than 0.6 eV. The XPS spectra of the initial surfaces were calibrated using the C1s binding energy of the carbon line adsorbed on the sample surface, assuming that the binding energy of the spectrum component corresponding to C–C/C–H bonds is 285.0 eV. The decomposition of the XPS spectra into components was carried out using the Avantage software. Before decomposing the spectra into components, Shirley background subtraction was used. The decomposition components had a symmetrical mixed form: 30% Lorenzian + 70% Gaussian.

To obtain the depth distribution of element concentrations, surface etching was used with a beam of Ar ions with an energy of 1 keV and an ion current density of 0.25 μA/mm^2^. The shooting of the XPS spectra after each subsequent 30 s etching of the sample surface allows for obtaining information on the distribution of elements in depth—i.e., dependence of the content of one or another element on the etching time. Since the surface modification process is associated with water electrolysis, the analysis of the results focused on the oxide components of the basic elements of the systems.

To study the effect of resistive switching, 4 ONSs of with sizes of 4 × 4 μm^2^ and thicknesses (sum of ONS height and PNS depth) from 0.8 ± 0.3 to 7.6 ± 0.6 nm were fabricated. Electrical measurements were carried out using a Ntegra built-in oscilloscope. The GaAs wafer was used as the lower contact; a cantilever ETHALON HA_NC with W_2_C coating was used as the upper contact. An alternating sawtooth sweep voltage with duration 100 ms and amplitude from 4 to 8 V for maximum R_HRS_/R_LRS_ ratio selecting was applied to the GaAs/ONS/W_2_C structures. As a result, 15 current-voltage (I-V) characteristics were obtained for each ONS. According to the results obtained, the dependences of the set voltage (U*_SET_*), resistances HRS and LRS, and HRS/LRS ratio on the ONS thickness were plotted.

## 3. Results and Discussion

### 3.1. Nanostructures

**Figure 2:**
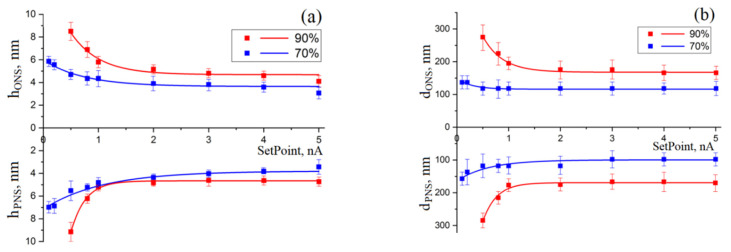
Dependences of the geometric parameters of nanostructures on the cantilever oscillation amplitude and the SetPoint parameter for the LAO surface of GaAs. The amplitude of the applied voltage is 15 V, the feedback circuit current is 2 nA, the air humidity is 1—90 ± 1% and 2—70 ± 1%: (**a**)—the height of the ONS and the depth of the PNS; (**b**)—the diameter of the ONS and PNS.

Analysis of the results obtained showed that an increase in humidity from 70 ± 1% to 90 ± 1% leads to an increase in the value of the SetPoint parameter, at which the LAO process begins, from 0.1 to 0.5 nA. An analysis of the dependences of the geometric parameters of ONS and PNS, formed by the LAO method on the SetPoint parameter (Figure 2) showed that an increase in SetPoint from 0.1 to 5 nA at a relative humidity of 70 ± 1% leads to a decrease in the PNS depth from 5.8 ± 0.4 to 3.1 ± 0.5 nm, in the ONS height from 6.9 ± 0.5 nm to 3.4 ± 0.6 nm, and in the diameter of ONS and PNS from 137.3 ± 20.1 to 98.3 ± 39.1 nm and from 157.7 ± 20.8 to 98.3 ± 20.2 nm, respectively.

Additionally, an increase in SetPoint from 0.5 to 5 nA at a relative humidity of 90% leads to a decrease in the ONS height from 8.5 ± 0.8 to 4.1 ± 0.5 nm, in the PNS depth from 9.1 ± 0.8 nm to 4.2 ± 0.3, as well as diameters ONS and PNS from 275.5 ± 58.2 to 137.3 ± 59.8 nm and from 284.5 ± 34.25 to 176.7 ± 29.3 nm, respectively. The decrease in the geometric parameters of oxide structures can be explained by the fact that an increase in the SetPoint parameter in the AFM semi-contact mode leads to an increase in the amplitude of cantilever oscillations, which, in turn, helps to reduce the time of interaction with the sample, decrease the rate of ONS formation and, accordingly, decrease the geometric dimensions of PNS gallium arsenide.

**Figure 3:**
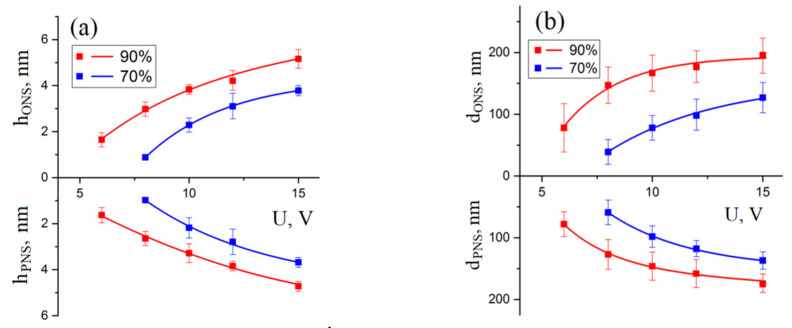
Dependences of the geometric parameters of nanostructures on the voltage amplitude, pulse duration 500 μs, feedback current 2 nA, air humidity 1—90 ± 1% and 2—70 ± 1%: (**a**)—the height of the ONS and the depth of the PNS; (**b**)—the diameter of the ONS and PNS.

An analysis of dependences of the geometric parameters of nanostructures on the LAO voltage amplitude (Figure 3) showed that, with an increase in relative humidity from 70 ± 1% to 90 ± 1%, the voltage amplitude decreases, at which point the LAO process occurs from 8.0 to 6.0 V. Relative humidity is one of the main parameters of the LAO process, characterizing the amount of water adsorbed on the surface of the substrate. The dependence of the threshold voltage of the LAO epitaxial structure of GaAs can be due to the number of water molecules in the probe-substrate system. At low relative humidity, the number of adsorbed water molecules is insufficient to ensure the oxidation of the substrate surface. Therefore, the threshold voltage at which the oxidation reaction begins will decrease with increasing relative humidity inside the technological SPM chamber. In addition, an increase in the geometric parameters of the ONS with an increase in relative humidity can occur due to an increase in the contact area between the probe and the water meniscus. Figure 3 shows that an increase in the amplitude of the voltage pulse leads to an increase in the geometric parameters formed by the ONS and PNS of GaAs. An increase in voltage from 8 to 15 V at a relative humidity of 70 ± 1% leads to an increase in the ONS height and PNS depth from 0.8 ± 0.1 to 3.7 ± 0.2 nm and from 0.9 ± 0.2 to 3.6 ± 0.2 nm, respectively. An increase in voltage from 6 to 15 V leads to an increase in the ONS height from 1.6 ± 0.3 to 6.3 ± 0.6 nm and an PNS depth from 1.6 ± 0.3 to 4.7 ± 0.7 nm at humidity 90 ± 1%. The diameter of the ONS and PNS on the surface of gallium arsenide also increased at a humidity of 70 ± 1% from 39.3 ± 20.7 to 127.4 ± 39.1 nm and from 59.3 ± 20.6 to 137.3 ± 27.4 nm, respectively. At a humidity of 90 ± 1%, the diameter of ONS and PNS increased from 78.3 ± 39.2 to 195.7 ± 39.2 nm and from 78.6 ± 20.1 to 175.7 ± 20.3 nm, respectively.

The dependences of the geometric parameters of the ONS and PNS on the voltage amplitude in LAO presented in Figure 2 can be explained as follows. An increase in the electric field leads to an increase in the number of active particles (oxygen ions and hydroxyl groups) formed as a result of the decomposition of water molecules in an electric field. Furthermore, the electric field directly affects the diffusion flux of active particles, which leads to an increase in the ONS growth rate [42].

**Figure 4:**
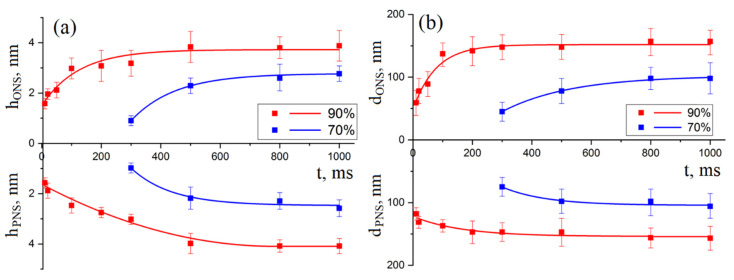
Dependences of the geometric parameters of nanostructures on the duration of the voltage. The amplitude of the applied voltage is 10 V, the feedback circuit current is 2 nA, the air humidity is 1—90 ± 1% and 2—70 ± 1%: (**a**)—the height of the ONS and the depth of the PNS; (**b**)—the diameter of the ONS and PNS.

An analysis of dependences of the geometric parameters of nanostructures on the LAO voltage duration (Figure 4) showed that, with an increase in humidity from 70 to 90 ± 1%, the pulse duration, at which the local anodic oxidation process begins, decreases from 300 to 10 ms. Figure 4 shows the experimental dependence of the height of the ONS and the depth of the PNS, as well as the diameter of the ONS and PNS on the duration of voltage pulses, obtained with a voltage amplitude of 10 V. Analysis of the results obtained showed that, at an increase in the pulse duration from 300 to 1000 ms at a relative humidity of 70 ± 1%, the ONS height and PNS depth increased from 0.9 ± 0.2 to 2.7 ± 0.3 nm and from 0.9 ± 0.2 to 2.5 ± 0.3 nm, respectively (Figure 4a). The diameter at a relative humidity of 70 ± 1% increased from 59.5 ± 20.8 to 157.1 ± 39.4 nm and from 118.3 ± 39.7 to 156.3 ± 19.6 nm for ONS and PNS, respectively (Figure 4b). An increase in the pulse duration from 10 to 1000 ms at a relative humidity of 90 ± 1%, the ONS height increases from 1.5 ± 0.2 to 3.8 ± 0.6 nm, and the PNS depth from 1.5 ± 0.2 to 4.1 ± 0.3 nm. The diameter at a relative humidity of 90% increased from 55.4 ± 15.2 to 153.5 ± 39.2 and from 113.8 ± 35.4 to 156.3 ± 24.6 nm for ONS and PNS, respectively.

**Figure 5:**
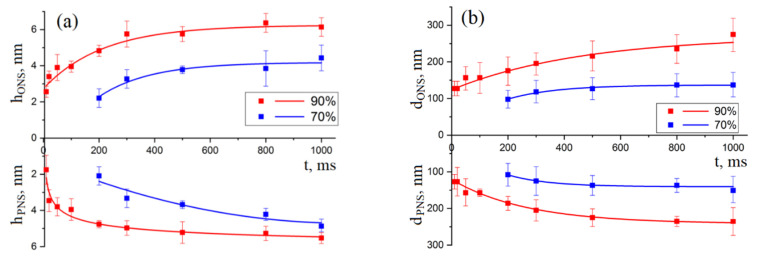
Dependences of the geometric parameters of nanostructures on the duration of the voltage. The amplitude of the applied voltage is 15 V, the feedback circuit current is 2 nA, the air humidity is 1—90 ± 1% and 2—70 ± 1%: (**a**)—the height of the ONS and the depth of the PNS; (**b**)—the diameter of the ONS and PNS.

Figure 5 shows the experimental dependence of the height of the ONS, the depth of the PNS, and the diameter of the ONS and PNS on the duration of the applied voltage pulse, obtained with a voltage amplitude of 15 V. An increase in pulse duration from 200 to 1000 ms at a relative humidity of 70 ± 1% leads to an increase the ONS height and the PNS depth from 2.2 ± 0.5 to 4.4 ± 0.7 nm and from 2.1 ± 0.5 to 4.8 ± 0.7 nm, respectively. The diameter at a relative humidity of 70 ± 1% increased from 98.3 ± 24.7 to 137.4 ± 39.1 nm and from 108.6 ± 31.3 to 151.6 ± 39.2 nm for ONS and PNS, respectively. An increase in the pulse duration from 10 to 1000 ms at a relative humidity of 90 ± 1% leads to an increase the ONS height from 3.9 ± 0.3 to 6.1 ± 0.5 nm, and the PNS depth from 1.8 ± 0.7 to 5.5 ± 0.3 nm. The diameter at a relative humidity of 90 ± 1% increased from 136.8 ± 58.8 to 275.3 ± 68.6 nm and from 137.2 ± 10.3 to 235.6 ± 59.2 nm for ONS and PNS, respectively. In addition, an increase in the voltage amplitude leads to the shifts of characteristics saturation from 495 ms at 10 V to 600 ms at 15 V, humidity 70 ± 1%; from 200 ms at 10 V to 600 ms at 15 V, humidity 90 ± 1%.

An analysis of Figure 3 and Figure 5 shows that the growth of GaAs ONS occurred more intensively at small values of the duration of voltage pulses. This can occur as a result of the diffusion of active particles to the surface of the epitaxial structure of GaAs through the formed ONS, whose thickness (Z_ONS_) at the beginning of the LAO process is insignificant; therefore, the ONS growth rate is high. With an increase in the Z_ONS_ of the formed ONS, the electric field in the oxide layer decreases, while the flow of active particles during LAO to the surface of the epitaxial structure of GaAs decreases, and, therefore, the speed of ONS formation decreases.

**Figure 6:**
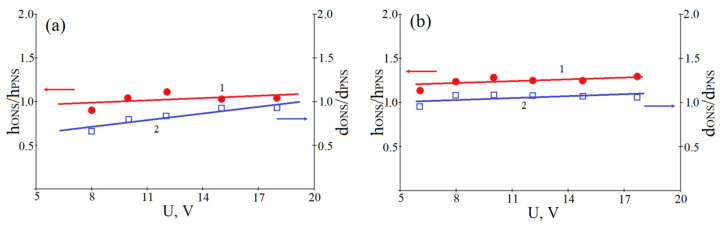
Dependence of the ratio of the height (diameter) of the ONS and the depth (diameter) of the PNS on the voltage amplitude of the LAO at different humidity: (**a**)—70 ± 1%; (**b**)—90 ± 1%. (1—ratio of ONS height and PNS depth; 2—ratio of ONS and PNS diameters).

Figure 6 shows the experimental studies of the dependences of h_ONS_/h_PNS_ and d_ONS_/d_PNS_ ratios on the voltage amplitude at relative humidity 70 ± 1% to 90 ± 1%. Analysis of the results obtained showed that the h_ONS_/h_PNS_ ratio is about 1.06 ± 0.12 at a relative humidity of 70 ± 1% and 1.10 ± 0.06 at a relative humidity of 90 ± 1%. In addition, the d_ONS_/d_PN_ ratio is about 0.82 ± 0.09 with a relative humidity of 70 ± 1% and 1.11 ± 0.04 with a relative humidity of 90 ± 1%. Thus, it was shown that, when the amplitude of the applied voltage changes, the h_ONS_/h_PNS_ and d_ONS_/d_PNS_ ratios do not change significantly.

**Figure 7:**
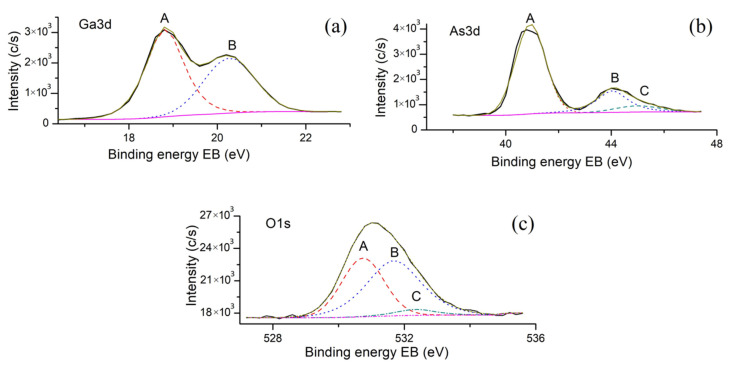
X-ray photoelectron Ga3d (**a**), As3d (**b**), O1s (**c**) spectra obtained for initial area of the GaAs surface.

Figure 7 shows the X-ray photoelectron O1s, Ga3d, and As3d spectra obtained from a modified area of the GaAs surface. An analysis of the obtained spectra shows a steady deficit of arsenic on the surface, exceeding the statistical error of the XPS method (<10%). As can be seen from the results, the Ga3d spectrum is decomposed into two components (A and B), the first of which (A) corresponds to Ga atoms embedded in the GaAs lattice, and the second (B) to atoms in Ga_2_O_3_ oxide. This indicates the absence of the effect of LAO modes on the phase composition of the GaAs ONS.

The As3d spectrum is decomposed into three components: the first one (A) corresponds to As atoms in the GaAs lattice, the second one (B) corresponds to As_2_O_3_, and the third one (C) corresponds to As_2_O_5_. The O1s spectrum is also decomposed into three components: the first one (A) corresponds to oxygen in the oxides Ga_2_O_3_ and As_2_O_3_, the second one (B) corresponds to oxygen in the oxide As_2_O_5_, and the third one (C) corresponds to adsorbed oxygen or hydroxyl. Thus, we can say that the formed oxide consists mainly of Ga_2_O_3_ and As_2_O_3_, since the contribution of the As_2_O_5_ phase is negligible due to its instability and tendency to dissolve in water [43].

**Figure 8:**
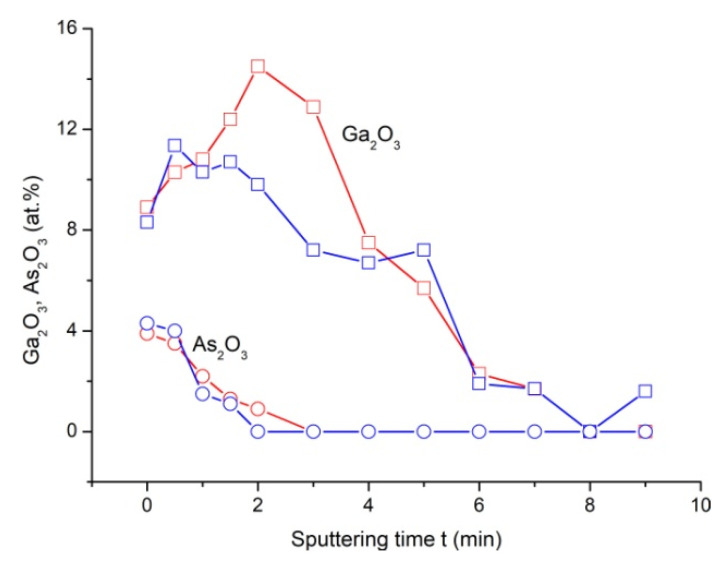
Dependences of the content of oxide components Ga_2_O_3_ (square symbols) and As_2_O_3_ (round symbols) in GaAs oxide on sputtering time: blue (symbols and lines)—for unmodified GaAs surface; red (symbols and lines)—for modified area.

Figure 8 shows the concentration profiles of components B of the Ga3d and As3d spectra corresponding to the Ga_2_O_3_ and As_2_O_3_ oxides. A comparison of the presented profiles shows that the surface concentration of As_2_O_3_ is two times lower than the surface concentration of Ga_2_O_3_. An increasing depth leads to As_2_O_3_ monotonical decrease to zero, while Ga_2_O_3_ increases to a maximum at some distance from the surface, and then decreases. In this case, the thickness of the Ga_2_O_3_ layer is 2–3 times greater than the thickness of the As_2_O_3_ area—i.e., the oxidized GaAs region consists mainly of Ga_2_O_3_.

Such a distribution of oxide phases over the oxide thickness can be associated with the possible reduction of arsenic oxides with H+ ions (with the formation of elemental arsenic) or their reaction with OH– hydroxide ions with subsequent dissolution of the products in an aqueous environment [43].

### 3.2. Effect of Resistive Switching

**Figure 9:**
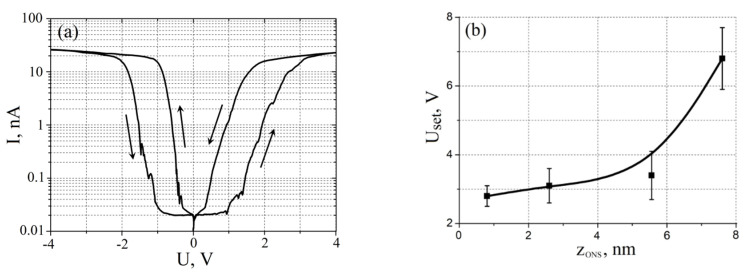
Investigation of resistive switching effect in of gallium arsenide ONS: (**a**)—*I-V* characteristic of ONS; (**b**)—*U_SET_* dependence on ONS thickness.

Figure 9 shows the results of experimental studies of the resistive switching effect in ONS gallium arsenide epitaxial structure. It is shown that the fabricated ONS’s exhibit a bipolar effect of resistive switching. An analysis of the results obtained shows that an increase in the ONS thickness from 0.8 ± 0.3 to 7.6 ± 0.6 nm leads to an increase in the switching voltage *Uset* from 2.8 ± 0.3 to 6.8 ± 0.9 V. The result can be explained by the fact that the formation of oxygen vacancies, which play a key role in resistive switching, requires energy above a certain threshold value determined by the oxide material. Thus, with an increase in the ONS thickness, to create the electric field strength at which the process of generating oxygen vacancies begins, it is necessary to increase the amplitude of the voltage applied to the GaAs/ONS/W_2_C structure [44,45].

**Figure 10:**
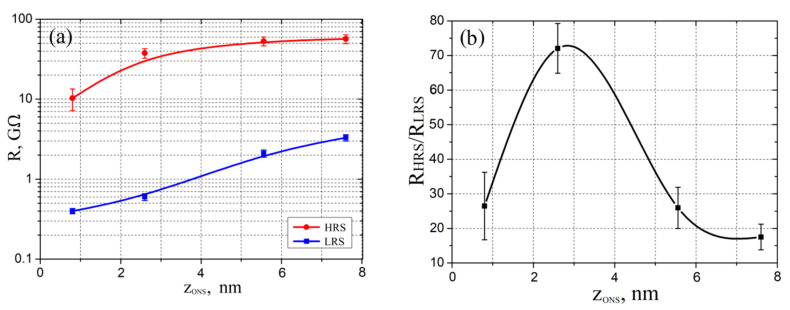
Dependences of ONS resistance on the thickness of ONS: (**a**)—*R_HRS_* and *R_LRS_*; (**b**)—*R_HRS_*/*R_LRS_*.

Figure 10 presents the experimental results of the influence of ONS thickness on the resistive switching effect in them. It was shown that an increase in the ONS thickness from 0.8 ± 0.3 to 7.6 ± 0.6 nm leads to an increase in *R_HRS_* from 10.32 ± 3.15 to 56.72 ± 7.17 GΩ, and *R_LRS_* from 0.41 ± 0.03 to 3.39 ± 0.31 GΩ (Figure 10a). The result can be explained by a decrease in current through the GaAs/ONS/W_2_C structure with an increase in the ONS thickness and, consequently, an increase in resistance. Additionally, analysis of the obtained results showed that an increase in the ONS thickness from 0.8 ± 0.3 to 2.6 ± 0.4 nm leads to an increase in the *R_HRS_/R_LRS_* ratio from 26.44 ± 9.76 to 72.05 ± 7.25, and to a decrease in the *R_HRS_/R_LRS_* ratio from 72.05 ± 7.25 to 17.52 ± 3.74 for thicknesses from 2.6 ± 0.4 to 7.6 ± 0.6 nm (Figure 10b). The result can be explained as follows: the ONS thickness, 0.8 ± 0.3 nm, is insufficient for the formation of a nanoscale conduction channel; therefore, resistance switching occurs due to a few oxygen vacancies. Therefore, the *R_HRS_/R_LRS_* ratio for ONS with a thickness of 0.8 ± 0.3 nm is not high and has large dispersion. A decrease in the *R_HRS_/R_LRS_* ratio with an increase in the ONS thickness from 2.6 ± 0.4 to 7.6 ± 0.6 nm can be explained by a decrease in the length of the destroyed portion of the nanoscale conduction channel.

From the XPS results, it is seen (Figure 8) that the fabricated ONSs have an inhomogeneous composition in depth, while the maximum concentration of Ga3d is observed in the gap between the surface of the ONS and the bottom of the ONS. It is known that the enthalpy of the formation of Ga_2_O_3_ is higher than the enthalpy of the formation of As_2_O_3_ (−1089.1 kJ/mol and −658.8 kJ/mol, respectively). From this it follows that, in the ONS region with the maximum concentration of Ga_2_O_3_, the energy of the formation of oxygen vacancies is of the highest in comparison with other sections of the ONS. If we assume that the length of the destroyed part of the conduction channel is limited by the surface of the ONS and the site with the maximum concentration of Ga_2_O_3_, and that the distance between the surface of the ONS and the site with the maximum concentration of Ga_2_O_3_ decreases with increasing thickness, it follows that the increase in the thickness of the ONS from 2.6 ± 0.4 to 7.6 ± 0.6 nm may be the reason for a decrease in the *R_HRS_/R_LRS_* ratio.

## 4. Conclusions

The paper presents the results of the fabrication of ONS and PNS on the surface of epitaxial structure of GaAs by local anodic oxidation. The LAO process is controlled by several basic operating parameters, such as: air humidity, amplitude, and duration of voltage pulses in the probe-substrate system, and cantilever oscillation amplitude. On the surface of the epitaxial structure of GaAs in the AFM semi-contact mode, test arrays of island ONSs were obtained. The results showed that the relative humidity of the atmosphere inside the technological chamber affects the voltage values, the duration of voltage pulses, and the SetPoint parameter, at which the local anodic oxidation process begins. In addition, with an increase in the amplitude and duration of impulses of the applied voltage during LAO, an increase in the height and diameter of the ONS occurs, as well as an increase in the depth and diameter of the PNS on the surface of the epitaxial structures of gallium arsenide. It was shown that the cantilever oscillation amplitude is also a controlling parameter of the LAO process; its increase contributes to a decrease in the height, depth, and diameter of oxide and shaped nanoscale structures. Thus, the ability to control the LAO process and the availability of proper software make it possible to form complex nanostructures that are used in nano-electronic devices, as well as in nano-photonics and other high-tech fields.

X-ray photoelectron spectra of ONS were obtained and studied. It was shown that an increasing depth leads to As_2_O_3_ monotonically decreasing to zero, while Ga_2_O_3_ increases to a maximum at distance from the surface and then decreases. The thickness of the Ga_2_O_3_ layer is 2–3 times greater than the thickness of As_2_O_3_ area—i.e., the oxidized GaAs region consists mainly of Ga_2_O_3_.

The experimental studies of the influence of ONS thickness on the resistive switching effect were obtained. It was shown that the fabricated ONSs exhibit a bipolar effect of resistive switching. An analysis of the results obtained showed that an increase in the ONS thickness from 0.8 ± 0.3 to 7.6 ± 0.6 nm leads to an increase in the switching voltage *Uset* from 2.8 ± 0.3 to 6.8 ± 0.9 V. An increase in the ONS thickness from 0.8 ± 0.3 to 2.6 ± 0.4 nm leads to an increase in the *R_HRS_/R_LRS_* ratio from 26.44 ± 9.76 to 72.05 ± 7.25, and to a decrease in the *R_HRS_/R_LRS_* ratio from 72.05 ± 7.25 to 17.52 ± 3.74 for thicknesses 2.6 ± 0.4 to 7.6 ± 0.6 nm.

This work demonstrates the possibility of creating ReRAM memristor structures based on epitaxial oxide structures of silicon doped gallium arsenide. The paper considers some experiments on the influence of local anodic oxidation regimes on the effect of resistive switching; however, for the fabrication of ReRAM, based on epitaxial oxide structures of gallium arsenide in the future, it is necessary to find solutions for many problems associated with the fabrication of an array of oxide memristor structures with specified geometric and electrophysical parameters in a single technological cycle: the study of the regularities of the formation of oxygen vacancies in the volume of epitaxial oxide structures of gallium arsenide; the study of number of switching cycles (endurance test) and the temporal stability of the resistive switching effect; the study of control parameters (voltage amplitude, voltage frequency, limiting current) on the parameters of the resistive switching effect.

The results can be used in the development of technological processes for the manufacturing of nano-electronic elements, such as ReRAM, as well as high-efficiency quantum dot lasers.
